# iCARE: An R package to build, validate and apply absolute risk models

**DOI:** 10.1371/journal.pone.0228198

**Published:** 2020-02-05

**Authors:** Parichoy Pal Choudhury, Paige Maas, Amber Wilcox, William Wheeler, Mark Brook, David Check, Montserrat Garcia-Closas, Nilanjan Chatterjee

**Affiliations:** 1 Department of Biostatistics, The Johns Hopkins University, Baltimore, MD, United States of America; 2 Division of Cancer Epidemiology and Genetics, National Cancer Institute, Rockville, MD, United States of America; 3 Department of Epidemiology, University of North Carolina at Chapel Hill, Chapel Hill, NC, United States of America; 4 Information Management Services, Silver Spring, MD, United States of America; 5 Division of Genetics and Epidemiology, Institute of Cancer Research, London, United Kingdom; 6 Department of Oncology, The Johns Hopkins University, Baltimore, MD, United States of America; McMaster University, CANADA

## Abstract

This report describes an R package, called the Individualized Coherent Absolute Risk Estimator (iCARE) tool, that allows researchers to build and evaluate models for absolute risk and apply them to estimate an individual’s risk of developing disease during a specified time interval based on a set of user defined input parameters. An attractive feature of the software is that it gives users flexibility to update models rapidly based on new knowledge on risk factors and tailor models to different populations by specifying three input arguments: a model for relative risk, an age-specific disease incidence rate and the distribution of risk factors for the population of interest. The tool can handle missing information on risk factors for individuals for whom risks are to be predicted using a coherent approach where all estimates are derived from a single model after appropriate model averaging. The software allows single nucleotide polymorphisms (SNPs) to be incorporated into the model using published odds ratios and allele frequencies. The validation component of the software implements the methods for evaluation of model calibration, discrimination and risk-stratification based on independent validation datasets. We provide an illustration of the utility of iCARE for building, validating and applying absolute risk models using breast cancer as an example.

## Introduction

Absolute risk models estimate disease risk in an upcoming time interval based on known risk factors for healthy individuals in a population, accounting for the presence of competing outcomes, such as death from other causes [1, 2]. Absolute risk models for cancers and other diseases have important clinical and public health applications. Assessment of absolute risk of disease is fundamental for developing health intervention strategies to optimize an individual’s risks and benefits. For example, absolute risk models can be used to identify individuals who have a high risk of disease in order to target screening and disease prevention strategies [3–6]. Decisions regarding the initiation of screening or preventive intervention are often made on the basis of age and family history, considered proxies for risk. However, there is increasing consensus in the medical community that these decisions should instead be guided directly by individualized estimates of risk, which can be obtained from absolute risk models that include a wider array of environmental and genetic risk factors. Assessment of the distribution of risks for individuals in the population allows public health researchers to weigh the risks and benefits of a given intervention, such as a screening regimen, for the entire population [7–9]. Absolute risk models can also be applied to assess the power of clinical trials by projecting the expected distribution of disease risk from the distribution of risk factors in a population [6]. At an individual level, absolute risk estimates can be used to counsel individuals on the basis of their personalized risk.

As large scale epidemiologic studies continue to discover new risk factors for many diseases, there is a growing demand to develop and apply models for absolute risk prediction that can facilitate translation of our understanding of etiology into tools for managing health. At present there does not exist a general software for researchers to build, update, evaluate and apply absolute risk models in R [10], and the Individualized Coherent Absolute Risk Estimation (iCARE) package provides this much needed capability.

The iCARE package builds absolute risk models by synthesizing multiple data sources containing information on relative risks, the distribution of risk factors in the population, and age-specific incidence rates for the disease of interest and rates of competing risks. This compartmentalization allows researchers to incorporate the best available information on key model parameters, to easily update models as new information becomes available, and to tailor or extend models to particular populations. For clinical applications of absolute risk prediction models, it is critical that they are validated in independent studies that did not contribute to the model building. Model validation involves two aspects: (i) model calibration i.e., whether the model is producing unbiased estimates of risk in subjects with varying risk factor profiles; (ii) model discrimination, i.e., ability of the model to draw distinction between cases and controls. We have updated iCARE with a validation component, that will allow evaluation of comparative performance of models, possibly across different studies, using a unified set of methods. Releasing iCARE will reduce that start up time for researchers, help standardize the methodology and make it easy to share absolute risk models and make associated analyses reproducible. The package also implements methods for handling missing covariate data, which is likely to be an issue in practice, and gives special attention to the efficient incorporation of genetic factors based solely on published information.

## iCARE methodology: Synthesizing data sources

Here, we present the statistical framework underlying the iCARE package. We describe the data inputs that are required to use the tool, examples of appropriate sources for the data, and details regarding how the key inputs are used to estimate model parameters. Specifically, we explain the methodology used to estimate the baseline hazard function component of the model and the approach used to handle missing data in the risk factor profiles used in the estimation of individuals’ risks. We describe the tool’s special treatment of SNPs, which allows genetic risk factors to be incorporated into the model based on published information.

### Model

The iCARE package fits a model for absolute risk, assuming the age-specific incidence rates of the disease given a set of risk factors, Z, follow the Cox proportional hazard (PH) model [11] of the form

$$\begin{array}{c} \text{Pr}\left(T\in \left[t,t+\Delta t\right)|T\geq t,Z\right)=\lambda \left(t|Z\right)=\lambda_{0}\left(t\right)\;\text{exp}\left(\beta^{T}Z\right), \end{array}$$

where *T* represents the time to onset of the disease of interest. The model assumes that risk factors *Z* act in a multiplicative fashion on the baseline hazard function, λ_0_(*t*). Given this model, the absolute risk of the disease for an individual who is currently at age *a* over the time internal *a* + *τ* is defined as [1],

$$\begin{array}{c} \int_{a}^{a+\tau }\lambda_{0}\left(t\right)\;\text{exp}\;\left(\beta^{T}Z\right)\;\text{exp}\;(-\int_{a}^{t}[\lambda_{0}\left(u\right)\;\text{exp}\;\left(\beta^{T}Z\right)+m\left(u\right)]du)dt. \end{array}$$ (1)

Formula (1) accounts for competing risks due to mortality from other causes through the age-specific mortality rate function *m*(*t*). In the current implementation, for simplicity, it is assumed that risk of mortality does not depend on the risk factor *Z*, but the method, in principle, can be extended to relax this assumption if covariate-specific risks of competing mortality can be estimated from external sources or models.

### Data and estimation

In order to build the above model and apply it for absolute risk estimation, users must provide three main data sources:

- a model for the log relative risk (or log hazard ratio) parameters: *β*
- a marginal age-specific disease incidence rate: λ_*m*_(*t*)
- a dataset containing risk factors for a set of representative individuals that could be used to estimate the risk factor distribution for the underlying population: *Z*_*j*_ for *j* = 1, …, *N*_*ref*_

In order to account for competing risks, an optional input with age-specific incidence rates of all-cause mortality, ideally excluding the disease of interest, *m*(*t*) should also be provided.

The iCARE tool computes absolute risk estimates as the sum of the integrand of (1) over integer ages in the time interval of interest. The user-provided log relative risk parameter estimates, $\hat{\beta }$, are plugged into the equation directly to carry out the computation. There are a number of ways that these input parameters may be obtained. For example, the estimates $\hat{\beta }$ may be derived from the analysis of a prospective cohort study using a multivariate PH model. It is recommended that assumptions, such as proportionality of hazards, are tested in those studies that produced the estimates of the regression coefficients and standard functions from R packages can be used for this step (e.g., survival package). Alternatively, they may be obtained from the analysis of a population-based incident case-control study using a multivariate logistic regression model adjusted for fine categories of age [12]. Ideally, datasets used to estimate model parameters should include information on all risk factors of interest and be large enough to provide precise estimates. When this is not available, estimates of relative risk for different risk factors could be obtained from multiple data sources (e.g., large published studies or meta-analyses). It is important that the provided estimates are adjusted for other risk factors in the model and interactions. Often information on odds-ratio parameter are more widely available in practice and one can approximate hazard ratios by odd-ratios under the assumption of rare disease, at least within small age intervals.

The second data source needed for the model is an estimate of the overall (or marginal) age-specific disease incidence rate, defined as

$$\begin{array}{c} \text{Pr}\left(T\in \left[t,t+\Delta t\right)|T\geq t\right)=\lambda_{m}\left(t\right), \end{array}$$

for the population of interest. This information, for example, could be available from population-based registries, such as the United States’ Surveillance Epidemiology and End Results (SEER) cancer registry maintained by the National Cancer Institute [13]. Similarly, users who wish to account for competing risks must provide the optional marginal age-specific incidence rates of all-cause mortality excluding the disease of interest

$$\begin{array}{c} \text{Pr}(M\in [t,t+\Delta t)|M\geq t)=m(t). \end{array}$$

In general it is best to incorporate rates defined for fine age categories, such as 1- or 5-year age strata, however iCARE can accommodate information on coarser age strata as well. For estimation, the age-specific disease incidence rates λ_*m*_(*t*) are used in combination with the third data input, a dataset of risk factors that is representative of the population of interest, to estimate the baseline hazard function, λ_0_(*t*).

### Estimating the baseline hazard function

Given the model of log relative risks, $\hat{\beta }$, and marginal age-specific disease incidence rates, ${\hat{\lambda }}_{m}\left(t\right)$, we use the following relationship to derive the baseline hazard rate

$$\begin{array}{c} \lambda_{m}\left(t\right)=\lambda_{0}\left(t\right)\text{E}[\text{exp}(\beta^{T}Z)|T\geq t)]=\lambda_{0}\left(t\right)\int \text{exp}\left(\beta^{T}z\right)\text{Pr}\left(z|T\geq t\right)dz, \end{array}$$ (2)

where, under the proportional hazard model,

$$\begin{array}{c} \text{Pr}\left(z|T\geq t\right)=\frac{\text{exp}(-\int_{0}^{t}\lambda_{0}\left(u\right)\;\text{exp}\left(\beta^{T}z\right)du)}{\int \;\text{exp}\;\{-\int_{0}^{t}\lambda_{0}\left(u\right)\;\text{exp}\left(\beta^{T}z\right)du\}dF\left(z\right)} \end{array}$$

with *F*(*Z*) denoting the distribution of the risk factors in the underlying population. If the disease can be assumed to be rare, then (2) can be approximated in closed form as

$$\begin{array}{c} \lambda_{m}\left(t\right)\approx \int \lambda_{0}\left(t\right)\;\text{exp}\left(\beta^{T}z\right)dF\left(z\right). \end{array}$$

Computationally, the iCARE implementation starts with an initial value for λ_0_(*t*) based on the rare disease approximation and iterates based on formula (2) to obtain more exact estimates. This approach is closely related to an alternative approach for estimation of λ_0_(*t*) described by [1]. The latter approach involves estimation of baseline hazard using the risk factor distribution from a random sample of cases. In contrast, our estimation method relies on an available distribution of the risk factors for a general population. Thus, a model based on our proposed method (as implemented by iCARE) can be easily updated to reflect the risk factor distribution for different populations without requiring access to a sample of cases from each population of interest.

### Specification of risk factor distribution

The risk factor distribution *F*(*Z*) plays a key role in calibrating the model to the marginal disease incidence rates in the underlying population. Thus, to carry out the calibration, the user must provide individual level data on the model risk factors for a sample that is representative of the underlying population. This multivariate dataset should reflect the correlations between the risk factors. Ideally, this representative dataset can come from a national survey, an epidemiologic study such as a population-based cohort or controls from a population-based case-control study sampled from the population of interest. When empirical data are available, there are no additional modeling assumptions needed. However, if complete empirical data in all risk factors are not available, users can instead provide a representative dataset that may have been simulated under modeling assumptions appropriate to the population of interest (e.g., independence of certain risk factors). We assume here that the user is going to use best possible resource(s) to build this dataset and feed that into the software.

### Handling missing data in covariate profile

In addition to providing the three data inputs for estimating model parameters, users must provide information on risk factors for the individuals to whom the model should be applied. When there is complete information for all risk factors of interest, risk estimation is as straightforward as plugging the risk factors *Z* into formula (1). However, in practice there may be missing data on some of the risk factors for individuals for whom we want to produce risk estimates.

One way to handle missing data on risk factors for a given individual is to use multiple imputation procedures [14]. The user would obtain estimates of absolute risk using iCARE for each of the completed-by-imputation risk factor profiles for the individual, and then average the absolute risk estimates to obtain an overall estimate of the absolute risk for that individual.

The iCARE tool also provides an internal option for handling missing data in the covariate profile for prediction: model-free imputation based on the reference dataset of risk factors provided by the user. The methodology underlying this imputation is as follows. For any subject indexed by *i* with a covariate profile *Z*_*i*_, we define the risk score *R*_*i*_ = *β*^*T*^ *Z*_*i*_, the linear predictor associated with the user specified log relative risk model. If a subject has missing values in some of the covariates, we partition $R_{i}=R_{iP}^{o}+R_{iP}^{u}$, where *P* indexes the observed pattern of missing data and where $R_{iP}^{o}={\beta_{P}^{o}}^{T}Z_{iP}^{o}$ and $R_{iP}^{u}={\beta_{P}^{u}}^{T}Z_{iP}^{u}$ denote the corresponding “observable” and “unobservable” components of the risk score. In general, this partitioning depends on which columns of the design matrix of the original model can be specified by the observed set of covariates for a given individual’s risk factor profile. Given this partitioning, the absolute risk, *AR*, of the individual *i* is defined by

$$\begin{array}{c} AR\left(R_{iP}^{o}\right)=\sum_{r_{iP}^{u}}AR\left(R_{iP}^{o},r_{iP}^{u}\right)\text{Pr}\left(r_{iP}^{u}|R_{iP}^{o}\right)=\text{E}[AR\left(R_{iP}^{o},R_{iP}^{u}\right)|R_{iP}^{o}]. \end{array}$$ (3)

The absolute risk for the *i*-th individual is obtained by averaging over possible values for the unobserved component of the risk score given the value of the observed component of the risk score. As all the risk scores are scalar quantities, one can estimate the conditional distributions $\text{Pr}(r_{iP}^{u}|R_{iP}^{o})$ in a fairly non-parametric fashion using the user-specified reference dataset.

In particular, to carry out (3) for a given covariate profile with missing data, the method finds subjects in the reference dataset that are similar to the individual with missing data on the basis of the observable component of the risk score, $R_{iP}^{o}$, informed by the observable risk factors. Specifically, the observable risk scores $R_{jP}^{o}$ are obtained for *j* = 1, ‥*N*_*ref*_ in the reference dataset, categorized into single percentile strata, and the individual’s observable risk-score $R_{iP}^{o}$ is matched to one of the strata. From the “matched” group, we then sample multiple times to fill in the unobserved part of the risk-score for the individual with missing data. The final absolute risk for an individual is calculated by taking average over the risks obtained from multiple filled-in values for the risk-score. So, this is essentially a model-free multiple imputation method where the user can specify the number of imputations (n.imps) and uncertainty is accounted for by taking average over risks from multiple filled in values for the risk-score. This imputation strategy based on the risk score can be viewed as a type of “hot deck” imputation in sample survey literature.

### Special option for SNP markers

As large genome-wide association studies continue to discover low penetrant, common SNPs associated with risk of complex chronic diseases, it is important to investigate the utility of the SNPs, in combination with other risk factors, for public health strategies of disease prevention. Evaluation of absolute risk, as opposed to relative risk which is typically used for summarizing associations, is fundamental for these public health applications. Due to the importance of SNP markers in absolute risk models and natural assumptions specific to genetic data, the iCARE package provides a number of options for incorporating SNPs into the model.

Users can include individual SNPs in the model, or include a polygenic risk score (PRS), in the same way as any other risk factor as long as all input components can be identified. This allows researchers to specify interactions between SNPs and other risk factors in the model or to include PRSs with more complex weighting structures, if desired. However, to include SNPs this way, a reference dataset must be provided that has the individual SNPs (or the PRSs) for all subjects. If the reference dataset is not available, the researchers may need to estimate this reference distribution by creating a simulated dataset of individuals who are representative of the underlying population.

Alternatively, the iCARE package also provides a special approach for handling independent SNPs, which requires that the user only provide information on the odds ratio *θ*_*k*_ and population allele frequency *f*_*k*_ for each SNP to be included. iCARE internally creates a PRS from all provided SNPs weighted by the odds ratios,

$$\begin{array}{c} PRS=\sum_{k}\text{log}\left(\theta_{k}\right)G_{k}, \end{array}$$

where *G*_*k*_ denotes the SNP genotype status of individuals, coded as the number of non-referent alleles they carry (with respect to the referent allele for which the odds ratios are reported).

In general, iCARE assumes this PRS to be distributed independently of all other covariates. However, if a family history (FH) variable is included in the model, then the method allows a simple adjustment to account for correlation between PRS and family history. The adjustment method assumes the latter is coded as a binary indicator of presence or absence of disease among first-degree relatives. In particular, when the model risk factors include family history, iCARE provides the option to adjust the log odds ratio associated with family history using the formula:

$$\begin{array}{c} \beta_{FH}^{A}=\beta_{FH}-0.5\sum_{k}\{\text{log}(\theta_{k}){\}}^{2}\times 2f_{k}\left(1-f_{k}\right) \end{array}$$

with *θ*_*k*_ denoting the disease odds ratio of the SNPs, unadjusted for family history. This adjustment reflects the fact that, with the addition of SNPs into the model, the effect of family history is attenuated by a magnitude that is proportional to the degree of heritability explained by the SNPs. This treatment should be applied only when the provided *β*_*FH*_ represents the association of a binary variable for presence or absence of disease among first-degree relatives, unadjusted for the SNPs. Users may provide relative risk estimates for family history that are already adjusted for the SNPs in the model, and if so they should simply not select the option for the family history adjustment.

One important way in which this approach treats SNPs differently involves the reference dataset of risk factors. This dataset might come from a national survey; however, a national survey is unlikely to have genotyped individuals, particularly for the exact set of SNPs to be included in the model. Recognizing this, for user convenience, the reference dataset just needs to include non-genetic risk factors and iCARE will simulate SNP genotype values based on the provided allele frequencies for the population. The SNPs are multiply imputed with user-specified number of imputations (n.imps) for each subject in the reference dataset. The method assumes that the SNPs are independent and that the genotype distributions follow Hardy-Weinberg Equilibrium in the population. Specifically, the joint distribution of SNP genotypes and other risk factors (*X*) are assumed to follow the decomposition

$$\begin{array}{c} \text{Pr}\left(g_{1},\ldots ,g_{k},X\right)=\text{Pr}\left(g_{1},\ldots ,g_{k}|FH\right)\times \text{Pr}\left(X\right). \end{array}$$

If family history of the disease is included in the model as a binary risk factor indicating the presence or absence of any first-degree relative with disease history, assuming that the disease is rare, we approximate

$$\begin{array}{c} \text{Pr}\left(g_{1},\ldots ,g_{k}|FH=0\right)\approx \text{Pr}\left(g_{1},\ldots ,g_{k}\right)=\text{Pr}\left(g_{1}\right)\times \ldots \times \text{Pr}\left(g_{k}\right). \end{array}$$

The distribution of SNP genotypes among subjects with family history is approximated as

$$\begin{array}{c} \text{Pr}\left(g_{1},\ldots ,g_{k}|FH=1\right)\approx \text{Pr}\left(g_{1}\right|FH=1)\times \ldots \times \text{Pr}\left(g_{k}|FH=1\right),\;\text{where}\\ \;\text{Pr}(g_{k}=g|FH=1)=\frac{\theta_{k}^{0.5g}\text{Pr}\left(g_{k}=g\right)}{\sum_{l=0}^{2}\theta_{k}^{0.5l}\text{Pr}\left(g_{k}=l\right)}. \end{array}$$

The above approximation is derived under the assumption of rare disease and multiplicative effect of SNPs on the risk of the disease and uses Mendel’s laws of inheritance. If family history is not indicated to be in the model, and is thus not provided for each reference dataset subject, we impute the SNPs based on the unconditional distribution for independent SNPs in Hardy-Weinberg equilibrium.

It is possible that SNP information may also be missing in the covariate profiles for whom the model will be applied to estimate risk. In this case, SNPs are treated the same as all other risk factors and handled according to the methodology as described earlier. This approach is equivalent to averaging over the possible values of the missing SNPs according to the population distribution, taking advantage of any known SNPs in the genotype profile.

### Computation of confidence intervals for absolute risk

Absolute risk estimation using iCARE requires information on the relative risk of the risk factors and population-based disease incidence rates and competing mortality rates and an individual level reference dataset of risk factors representative of the underlying population. One can compute confidence intervals for absolute risk using the bootstrap methods. We can assume that the incidence rates and competing mortality rates to be known without uncertainty. The relative risks can be re-sampled using a parametric bootstrap approach assuming a multivariate normal distribution with the mean and variance covariance matrix derived from prior studies. The reference dataset can also be re-sampled using non-parametric bootstrap. For each sample, we can estimate the absolute risk using the formula (1). This will give a distribution of the absolute risks based on the bootstrapped re-samples and the confidence intervals can be computed using this distribution. Given the formula (1) of absolute risk is a smooth functional of the different input parameters (relative-risk and reference risk-factor distribution), we expect from standard theory that this will be a valid method.

In risk prediction literature, there is debate about the value of uncertainty estimates associated with individual risk estimates beyond measures. The risk itself is a measure of uncertainty associated with whether one individual will develop a disease in the future or not and a concept clinicians and general public often struggle with. While providing an estimate of uncertainty of uncertainty can be of some statistical interest, how such information can be useful in practice is unclear. Like other popular risk tools (e.g., the Gail model: https://bcrisktool.cancer.gov), the current version of iCARE does not return estimate of uncertainty, such as 95% CI, with the estimate of the absolute risk of the individual.

### Model validation methods

Suppose we are given a model to predict the absolute risk of a disease. In other words, the different pieces of information needed to estimate absolute risk in (1) are given to us. Moreover, we also have an independent prospective cohort study (or a case-control study nested within a cohort), that we would like to use to validate the absolute risk model. Given below are the steps for implementing the model validation methods in the independent study.

#### Model calibration

Model calibration attempts to answer the question whether the risk prediction model is producing unbiased estimate of risk for subjects with varying risk factor profiles. The observed followup for a subject in the validation cohort is defined as the time from study entry to the last contact or linkage for acquiring information. It is possible to validate a model over the entire follow-up period of study. However, researchers often desire to validate a model for absolute risk over a fixed year (*τ*) of follow-up (e.g., 5-year or 10-year). In that scenario, the predicted risk for each subject is calculated over the minimum of *τ* years and observed follow-up. The predicted risk can be supplied by the user. Alternatively, it can be computed by iCARE, using Eq (1), with the input components supplied by the user.

For evaluating model calibration, the subjects who developed the disease over the minimum of *τ* years and observed follow-up were considered as cases and the subjects who did not were designated as controls. We categorize the validation cohort based on low risk to high risk of disease based on a pre-specified number of categories (e.g., deciles) of the risk score *R*_*i*_. We denote the categorical version of risk score as ${\tilde{R}}_{i}$. The categories are indexed by *c* = 1, …, *C* (e.g., C = 10 for deciles). At each category, we compute the observed proportion of cases (${\hat{p}}_{o,c}$) in that category. For comparison, we also compute an average of the subject specific predicted absolute risk of disease, ${\hat{p}}_{e,c}$, over the subjects in that category. An influence function based variance formula for ${\hat{p}}_{o,c}$ is implemented (details in Appendix). The 95% Wald based confidence intervals for ${\hat{p}}_{o,c}$ are computed using this variance formula. iCARE reports a ratio of the expected to observed number of cases and its 95% confidence interval. The popular Hosmer-Lemeshow type test for goodness of fit is also implemented, where the test statistic has the form:

$$\begin{array}{c} HL=\sum_{c=1}^{C}\frac{{\left({\hat{p}}_{o,c}-{\hat{p}}_{e,c}\right)}^{2}}{\text{Var}[{\hat{p}}_{o,c}]} \end{array}$$

The above HL statistic approximately follows a chisquare distribution with *C* degrees of freedom. This test, in spite of its limitations [15], is intuitively appealing and easily understood by subject matter scientists. The next version of iCARE will implement more sensitive tests for model validation based on the approach proposed in [15] and the R package rms.

The software also has capabilities to perform calibration on the scale of relative risks. The observed relative risk in each category is defined as the ratio of the observed proportion of cases in that category and the observed proportion of cases in the validation study. For comparison, the predicted relative risk is computed as the average of the predicted absolute risk over the subjects in that category and the overall average of the predicted absolute risks in the validation study. A variance formula for the observed relative risk is implemented (details in Appendix) and is used to compute the 95% Wald based confidence intervals. A chi-square goodness of fit test for relative risk is also implemented. It is possible to check model assumptions (e.g., proportional hazards assumption) by investigating relative risk calibration in different categories of age.

In a cohort study, data on some expensive bio-markers (e.g., genetic risk factors) are often collected only on a judiciously selected sub-sample nested within the original cohort (e.g., a nested case-control sample). In this case, the estimates of absolute and relative risks need to be adjusted to account for the non-random sampling using sampling weights. An inverse-probability weighted (IPW) approach is implemented to estimate the absolute risks. Let *I*_*i*_ denote the indicator of inclusion of a cohort subject (indexed by *i*) to the nested case-control sample and *Y*_*i*_ denote the case-control status. Denote by *π*_*i*_(*Y*_*i*_, *Z*_*i*_) = *Pr*[*I*_*i*_ = 1|*Y*_*i*_, *Z*_*i*_], the sampling weight, i.e., the probability of inclusion for a subject indexed by *i*. The cut-points for the risk score categories are calculated using these sampling weights by inverting a weighted empirical distribution function of the risk score. The adjusted estimates of observed proportion of cases and average of predicted risks, overall and at each category, are computed as the corresponding weighted averages (instead of simple averages) with weights being inverse of the sampling weights. A variance estimator of the observed proportion of cases in each category is also implemented using the influence function approach (details in Appendix). The 95% Wald based confidence interval is computed using this variance formula. The Hosmer-Lemeshow type goodness of fit test statistic also uses this variance formula in its denominator.

#### Model discrimination

For well calibrated models, it is critical that the risk factors used in the model have good discriminatory power i.e., ability to differentiate between cases and controls. A risk prediction model with good discriminatory ability ensures that there is a good amount of spread in the risk distribution of the subjects to facilitate risk stratification leading to better evaluation of risks and benefits. A common measure of model discrimination is the Area Under the Curve (AUC) defined as *δ* = *Pr*[*R*_1_ > *R*_0_], i.e., the probability that the risk score for a case (*R*_1_) is larger than the risk score for a control (*R*_0_). For a cohort study, this probability can be estimated using the empirical proportion of case-control pairs for which the risk score of the case is greater than that of the control. For this setting, the asymptotic variance formula of this estimator [16] is implemented and used to compute the 95% Wald based confidence interval. For a nested case-control study, we implement an inverse probability weighted estimator of AUC [17–21]. We also implement an influence function based variance estimate. The influence function representation and the variance formula are shown in the Appendix and further details are provided in [22]. The 95% Wald based confidence interval of this estimator is computed using this variance estimate.

## Existing R packages

In this section, we describe some of the unique aspects of iCARE compared to existing R packages for building and validating a model for predicting risk of a disease (see Table 1 for a summary). There are additional software packages developed in other platforms that allow risk calculation of specific cancers, e.g., IBIS Breast Cancer Risk Evaluation Tool [23, 24] and Breast and Ovarian Analysis of Disease Incidence and Carrier Estimation Algorithm (BOADICEA) [25–28].

**Table 1:** Summary of features of existing R packages for risk prediction. ✓ denotes the presence of the feature and × denotes absence of the feature.

Packages	Model building				Model validation		
	Calibration to population incidence	Detailed family history	Special option for SNP markers	Imputation of missing risk-factors	Full cohort	Two-phase study	Imputation of missing risk-factors
riskRegression^a^	×	×	×	×	✓	×	×
predictABEL^a^	×	×	×	×	✓	×	×
BCRA^b^	✓^c^	×	×	×	×	×	×
BayesMendel^b^	×	✓	×	✓	×	×	×^f^
rmap	×	×	×	×	✓	✓^e^	×
iCARE ^b^	✓^c^	×	✓^d^	✓	✓	✓^e^	✓^f^

^a^ These packages include some functions for model building (see Section), but those approaches do not demonstrate the key features shown in the above table.

^b^ Capability to use information from multiple data sources, e.g., BCRA and iCARE can use relative risk parameters from cohort or case-control studies and disease incidence and mortality rates from population registries.

^c^ BCRA estimates baseline hazard and calibrates the model to the underlying population incidence rates using distribution of risk-factors from cases in a specific study that may not be representative of the general population. This step is implemented in iCARE using a reference dataset that provides information on the distribution of risk-factors in the general population.

^d^ iCARE includes the special option in which independent SNP markers can be included using published estimates of odds ratios and allele frequencies.

^e^ Inverse probability weighted estimators of model validation statistics are implemented, accounting for bias due to non-random sampling using sampling weights.

^f^ BayesMendel incorporates imputation methods for certain risk-factors (e.g., age), but they do not implement any method of validating risk prediction models. iCARE implements an inbuilt imputation approach to deal with missing risk-factors using a reference risk-factor dataset representative of the underlying population. The standardized model validation methods implemented in iCARE can take advantage of this inbuilt feature to impute missing risk-factors in the validation study.

### Model building

The riskRegression package [29–31] implements various regression based methods for estimation of cumulative incidence in a cohort study setting with time-to-event outcome subject to censoring. The methods cannot be easily adapted to use information from multiple data sources, such as relative risk parameters from case-control studies and disease incidence information from population registries. In the PredictABEL package [32], the conditional probability of disease given risk factors is estimated using a logistic regression model. The target quantity is different from absolute risk. The BayesMendel package [33] implements Bayesian approaches to genetic risk prediction using information from detailed family history, genetic test results and some tumor characteristics. It is based on Mendelian inheritance mechanisms in the setting of family based studies of some specific cancers (e.g., breast cancer, colorectal cancer, pancreatic cancer, endometrial cancer). As opposed to their method, we focus on risk prediction in the general population using information from common genetic variants and epidemiologic risk factors in settings where detailed family history may not be available.

The BCRA package [34] is developed to calculate risk projections for invasive breast cancer based on the Gail model [1], and its subsequent extensions in the different ethnic groups [35–38]. The model uses information from some epidemiologic risk factors and family history of breast cancer. The basic setup of iCARE and BCRA are similar as both build models for absolute risk using information from different data sources and they use the same formula for the absolute risk based on an underlying Cox proportional hazard model [1]. There are, however, important differences in the underlying methodology. For estimating baseline hazard and calibrating the model to the underlying population incidence rates, BCRA uses the distribution of risk factors in cases of the study population. In contrast, we perform this calibration step using the distribution of risk factors coming from the general population. Our approach allows for developing risk models that reflect the distribution of risk factors in the underlying population without having to use the distribution of risk factors from cases of a specific study that may not be representative of the general population. While the BCRA does not have an inbuilt machinery to handle missing risk-factor data, the iCARE uses a coherent approach that involves internal imputation of missing risk-factors from the same model with appropriate model averaging.

### Model validation

The riskRegression and PredictABEL packages have limited capability of model validation in a cohort study setting. The rmap package [39–41] implements methods for independent evaluation of risk prediction models in studies that may involve two phase designs. One unique feature of iCARE, that is not available in other packages, is that it includes an exhaustive set of functions so that model building and model validation can be implemented in tandem. Thus the iCARE based model validation procedures can take advantage of the inbuilt features of the package to deal with summary level genetic information and imputation of missing risk factors.

## Illustrations

In this section, we demonstrate how to use iCARE to build and apply two absolute risk models for breast cancer: one with only SNP markers, and one with classical risk factors and SNPs. We also illustrate the use of the validation component of the package.

We use computeAbsoluteRisk function in iCARE to compute absolute risk. The input arguments to this function are named with the prefix “model.” or “apply.” according to whether they are used primarily for model building or application respectively.

The R package can be downloaded from the GitHub repository (https://github.com/parichoy/iCARE) using the commands:

R> install.packages(“devtools”, dependencies = TRUE)

R> library(devtools)

R> devtools::install_github(“parichoy/iCARE”)

Alternatively, it can be downloaded and installed from the Bioconductor website (http://bioconductor.org/packages/release/bioc/html/iCARE.html) using the instructions provided there. The GitHub version of the package is non-archival and may acquire new features earlier, while the Bioconductor version is archival.

In the next step, we load the package and the example dataset object bc_data:

R> library(“iCARE”)

R> data(“bc_data”, package = “iCARE”)

R> set.seed(50)

### Example 1: SNP-only model

To specify a SNP-only model, we must input the marginal age-specific incidence rates of breast cancer, bc_inc, and the SNP information matrix, bc_72_snps, that has three columns named: snp.name, snp.odds.ratio, and snp.freq. Marginal age-specific incidence rates of competing risks (mort_inc) are optional. We include them in this example.

Here, bc_72_snps contains published information on odds-ratios and allele frequencies of 72 SNPs identified, among a larger set of markers, to be associated with breast cancer risk by a recent genome-wide association study [42]. bc_inc contains age-specific incidence rates of breast cancer from Surveillance, Epidemiology and End Results (SEER) Program, and mort_inc has age-specific incidence rates of all-cause mortality from the WONDER mortality database [43]. In fitting a SNP-only model, the reference dataset need not be provided as iCARE will impute the reference SNP distribution based on SNP allele frequencies. The function call below builds an absolute risk model based on 72 SNPs for breast cancer and applies the model to estimate risk of breast cancer in the interval from age 50 to age 80:

R> res_snps_miss = computeAbsoluteRisk(model.snp.info = bc_72_snps,

            model.disease.incidence.rates = bc_inc,

            model.competing.incidence.rates = mort_inc,

            apply.age.start = 50,

            apply.age.interval.length = 30,

            return.refs.risk = TRUE)

For this SNP-only model, we first opted for not providing any new profiles for estimation (i.e., no apply.snp.profile input). In this case, iCARE simulates 10,000 SNP profiles internally for the reference dataset and reports as the risk estimate the average of the risks estimated from the profiles: 0.096. We can access the estimated risks for the (simulated) referent profiles and obtain summary information by calling:

> summary(res_snps_miss$refs.risk)

  Min. 1st Qu. Median   Mean 3rd Qu.   Max.

0.05745 0.08666 0.09494 0.09600 0.10422 0.15882

From this, we learn that on average women of age 50 years have a 9.6% chance of being diagnosed with breast cancer before age 80, and that the 72-SNP model stratifies breast cancer risk from a minimum risk of 5.75% to a maximum risk of 15.88% in the age interval 50 years to 80 years.

Next, suppose we want to predict risk for three specific women whom we have genotyped; we can then call:

R> res_snps_dat = computeAbsoluteRisk(model.snp.info = bc_72_snps,

            model.disease.incidence.rates = bc_inc,

            model.competing.incidence.rates = mort_inc,

            apply.age.start = 50,

            apply.age.interval.length = 30,

            apply.snp.profile = new_snp_prof,

            return.refs.risk = TRUE)

**Fig 1:**
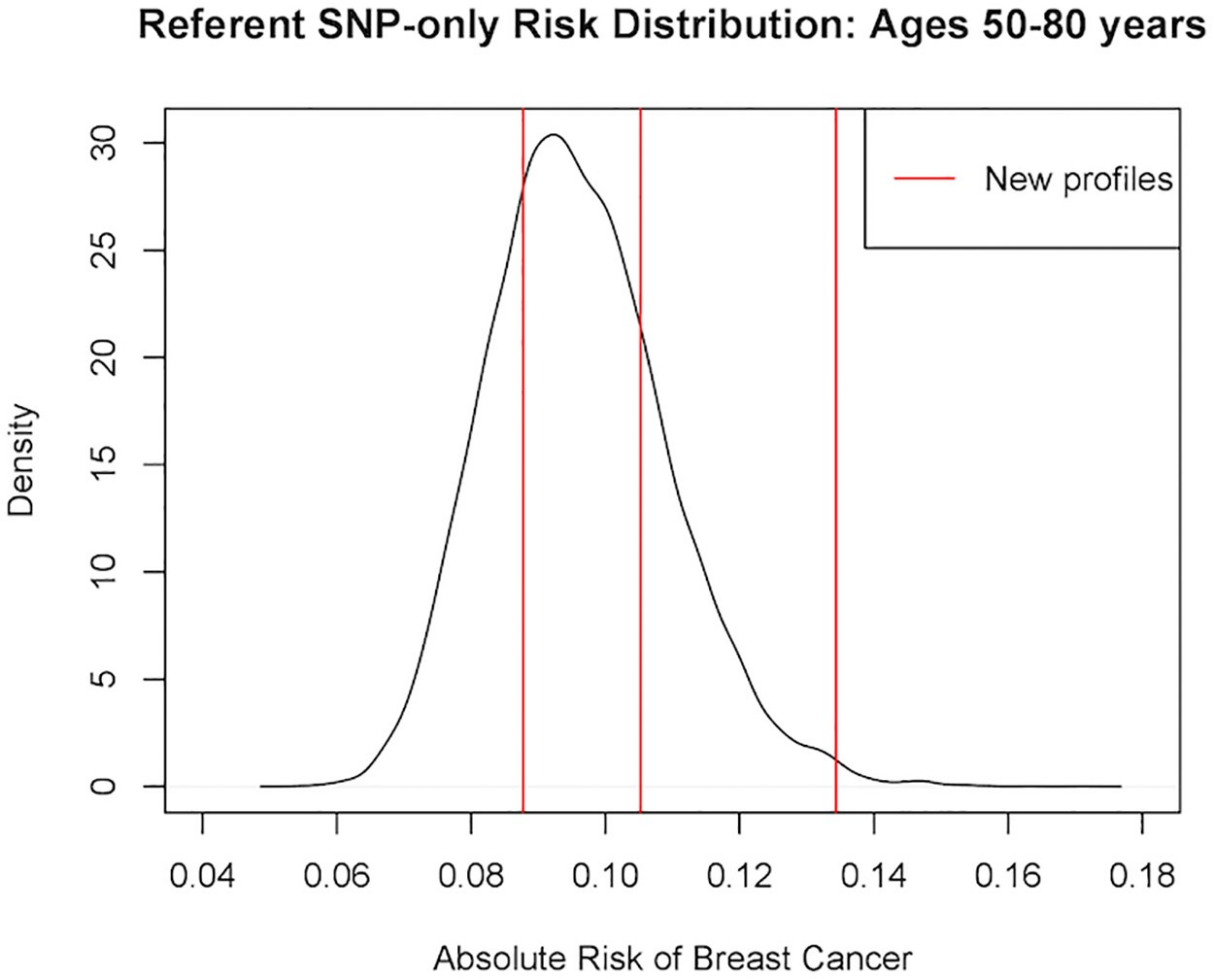
Estimated absolute risk of three women overlaid on the population distribution of absolute risk in the age interval: 50-80 years.

Now our output res_snps_dat$risk contains the risk estimates for the three women whose genotype profiles were provided in apply.snp.profile = new_snp_prof. Additionally, we have put the option: return.refs.risk = TRUE. Hence, res_snps_dat$refs.risk returns the risk estimates for the reference dataset based on 10,000 internal simulations. These results allow us to create a useful plot (Fig 1) showing the distribution of risks in our reference dataset and to add the risks of the three women to see where they fall on the population distribution. We use the code:

R> plot(density(res_snps_dat$refs.risk),

    xlim = c(0.04,0.18), xlab = “Absolute Risk of Breast Cancer”,

    main = “Referent SNP-only Risk Distribution: Ages 50-80 years”)

R> abline(v = res_snps_dat$risk, col = “red”)

R> legend(“topright”, legend = “New profiles”, col = “red”, lwd = 1)

In this example, the first genotype profile had missing data on five SNPs. The second genotype profile had missing data on a different set of five SNPs. This demonstrates the capability of iCARE to produce risk estimates when there is missing data in the risk factor profile without compromising on user convenience.

### Example 2: Breast cancer risk model with risk factors and SNPs

In this example, we illustrate the use of iCARE to build models of absolute risk with both classical risk factors and SNPs. The classical risk factors included in the model are shown in Table 2 [44].

**Table 2:** Detailed information about the classical risk factors included in the model.

Risk factor	Variable name	Variable type
Family history (presence or absence of disease among first degree relatives)	famhist	Binary
		Presence
		Absence
Age at menarche (years)	menarche_dec	Categorical:
		≤ 11
		11-11.5
		11.5-12
		12-13
		13-14
		14-15
		>15
Parity (number of full term pregnancies)	parity	Categorical
		nulliparous
		1 birth
		2 births
		3 births
		≥ 4 births
Age at first birth (years)	birth_dec	Categorical
		≤ 19
		19-22
		22-23
		23-25
		25-27
		27-30
		30-34
		34-38
		>38
Age at menopause (years)	agemeno_dec	Categorical
		≤40
		40-45
		45-47
		47-48
		48-50
		50-51
		51-52
		52-53
		53-55
		>55
Height (meters)	height_dec	Categorical
		≤ 1.55
		1.55-1.57
		1.57-1.60
		1.60-1.61
		1.61-1.63
		1.63-1.65
		1.65-1.66
		1.66-1.68
		1.68-1.71
		>1.71
Body mass index (BMI, *kg*/*m*^2^)	bmi_dec	Categorical
		≤ 21.5
		21.5-23
		23-24.2
		24.2-25.3
		25.3-26.5
		26.5-27.8
		27.8-29.3
		29.3-31.4
		31.4-34.6
		>34.6
Use of Hormone Replacement Therapy (HRT)	rd_menohrt	Categorical
		Premenopausal
		Postmenopausal and never HRT user
		Postmenopausal and ever HRT user
Use of estrogen + progesterone combined therapy	rd2_everhrt_c	Binary
		Postmenopausal and ever user of combined therapy therapy
		Otherwise
Use of estrogen only therapy	rd2_everhrt_e	Binary
		Postmenopausal and ever user of estrogen only therapy
		Otherwise
Current use of HRT	rd2_currhrt	Binary
		Postmenopausal and current HRT user
		Otherwise
Alcohol, drinks/week	alcoholdweek_dec	Categorical
		None
		0-0.4
		0.4-0.8
		0.8-1.5
		1.5-3.2
		3.2-5.7
		5.7-9.8
		>9.8
Smoking status	ever_smoke	Binary
		Ever
		Never
Interaction of BMI and use of HRT	rd_menohrt*bmi_dec	Categorical

The first step is to prepare the input parameters bc_model_cov_info, containing the classical risk factor information and bc_model_formula, containing the model formula object for the part of the model with epidemiologic risk factors only.

After preparing the data sources, we can now run:

R> res_covs_snps = computeAbsoluteRisk(model.formula = bc_model_formula,

                model.cov.info = bc_model_cov_info,

                model.snp.info = bc_72_snps,

                model.log.RR = bc_model_log_or,

                model.ref.dataset = ref_cov_dat,

                model.disease.incidence.rates = bc_inc,

                model.competing.incidence.rates = mort_inc,

                model.bin.fh.name = “famhist”,

                apply.age.start = 50,

                apply.age.interval.length = 30,

                apply.cov.profile = new_cov_prof,

                apply.snp.profile = new_snp_prof,

                return.refs.risk = TRUE)

We indicate model.bin.fh.name = “famhist” to allow the software to properly attenuate the log odds ratio for family history to account for the addition of the 72 SNPs. With the exception of model.bin.fh.name, which is always optional, the arguments model.formula, model.cov.info, model.log.RR, model.ref.dataset, apply.cov.profile should either be included or excluded in the function call as a set. This is to say that if one is included, then all should be included.

The above function call fits an absolute risk model with classical risk factors and 72 SNPs associated with breast cancer. In a model that includes risk factors, such as this one, we must supply the model formula, the risk factor information, the log odds ratios for the risk factors, and a reference dataset of risk factors to build the model. The model.cov.info gives the variable names and types (i.e., continuous or categorical) for the classical risk factors. The bc_model_log_or input contains the log odds ratios for classical risk factors, from a logistic regression model adjusted for cohort and fine categories of age in the Breast and Prostate Cancer Cohort Consortium [45–47]. The ref_cov_dat dataset was created by simulation from the National Health Interview Survey (NHIS) and the National Health and Nutrition Examination Survey (NHANES), which are representative of the US population.

In addition to summarizing and plotting the risk estimates, iCARE includes an option to view more detailed output, by calling:

R> print(res_covs_snps$details)

This reports the interval start and end ages over which absolute risk was computed, the entire covariate profile to which the model was applied (SNPs and risk factors, if applicable), and the resulting risk estimate.

In this example, some of the classical risk factors were specified as decile categories to model parsimoniously the non-linearity asscoiated with certain risk factors as reported in the paper by Maas *et al*. [44]. It is possible to specify as input parameters in iCARE, more complex model fit that may include non-linear transformations (e.g., splines) of continuous predictors. The software has capabilities to generate absolute risks based on such models provided the user appropriately specifies the input parameters: model formula, model covariate information, log-relative risks, variables for the reference dataset.

### Validation component

We illustrate the working of the model validation component of iCARE by evaluating a risk prediction model with both classical risk factors and SNPs using simulated data.

The package includes a full cohort data in validation.cohort.data generated through simulation. There are 50,000 subjects with information on age of study entry, age of study exit, time to disease onset, disease status and classical risk factors in Table 2. A common scenario in modern cohort studies is that the genetic factors are not measured on all the subjects in the cohort, but only on a judiciously selected sub-sample. The package also includes a case-control sample (validation.nested.case.control.data) of 5,285 women nested within the full cohort with information on age of study entry, age of study exit, time to disease onset, disease status, classical risk factors and 72 breast cancer associated SNPs. Further details on how the full cohort and nested case-control data were simulated are provided in the Appendix.

To validate a model with epidemiologic risk factors and SNPs, we will use the nested case-control sample. The calibration and discrimination statistics need to be adjusted for the non-random sampling using sampling weights (i.e., probability of selection of a subject to the nested case-control sample). When sampling weights are not provided, we recommend using post-hoc estimation by assuming parametric models for selection mechanism. We illustrate the sampling weight calculation through a logistic regression model of inclusion on disease status, age of study entry, observed follow-up and interaction of disease status with age of study entry and observed follow-up.

The first step is to identify the subjects in the full cohort who are included in the nested sub-sample through matching of subject identifiers and create the indicator of inclusion:

R> validation.cohort.data$inclusion = 0

R> subjects_included = intersect(validation.cohort.data$id,

                 validation.nested.case.control.data$id)

R> validation.cohort.data$inclusion[subjects_included] = 1

The next step is to fit a logistic regression model of inclusion depending on the case/control status, age of study entry and observed followup using the R function glm, as shown below:

R> validation.cohort.data$observed.followup =

              validation.cohort.data$study.exit.age -

                 validation.cohort.data$study.entry.age

R> selection.model = glm(inclusion ~ observed.outcome

             * (study.entry.age + observed.followup),

                 data = validation.cohort.data,

                 family = binomial(link = “logit”))

R> validation.nested.case.control.data$sampling.weights =

   selection.model$fitted.values[validation.cohort.data$inclusion == 1]

In the above code chunk, the second line shows the call to the glm function to fit the selection model. Once we have the sampling weights, we can call the ModelValidation function in iCARE that implements the validation analysis. We specify the risk prediction model formula with epidemiologic risk factors only in bc_model_formula. The list object risk.model combines all the input parameters required by the computeAbsoluteRisk() function, used internally by the ModelValidation function to estimate absolute risks in the validation study.

R> data = validation.nested.case.control.data

R> risk.model = list(model.formula = bc_model_formula,

        model.cov.info = bc_model_cov_info,

        model.snp.info = bc_72_snps,

        model.log.RR = bc_model_log_or,

        model.ref.dataset = ref_cov_dat,

        model.ref.dataset.weights = NULL,

        model.disease.incidence.rates = bc_inc,

        model.competing.incidence.rates = mort_inc,

        model.bin.fh.name = “famhist”,

        apply.cov.profile = data[,all.vars(bc_model_formula)[-1]],

        apply.snp.profile = data[,bc_72_snps$snp.name],

        n.imp = 5, use.c.code = 1,

        return.lp = TRUE, return.refs.risk = TRUE)

R> output = ModelValidation(study.data = data,

               total.followup.validation = TRUE,

               predicted.risk.interval = NULL,

               iCARE.model.object = risk.model,

               number.of.percentiles = 10)

In the above function call, the argument iCARE.model.object stores the list of input parameters that are used by iCARE to build an absolute risk model and compute model based estimates of risk in the validation study. The arguments study.data inputs the nested case-control sample used for model validation. In this example, we have set total.followup.validation = TRUE and predicted.risk.interval = NULL to indicate that we are validating the risk over the duration of follow-up of the subjects in the validation study. One can set these parameters appropriately to validate *τ*-year risk in a given study. We have set number.of.percentiles = 10 to indicate that the categories of low risk to high risk of disease were defined based on the deciles of the risk score.

The output of model validation can be viewed using the print command as follows:

> print(output)

Dataset: Example Dataset

Model Name: Example Risk Prediction Model

Model Formula: diagnosis ~ famhist + as.factor(menarche_dec) +

                as.factor(parity) + as.factor(birth_dec) +

                as.factor(agemeno_dec) +

                as.factor(height_dec) +

                as.factor(bmi_dec) + as.factor(rd_menohrt) +

                rd2_everhrt_e + rd2_everhrt_c + rd2_currhrt +

                as.factor(alcoholdweek_dec) +

                as.factor(ever_smoke) +

                as.factor(rd_menohrt) * as.factor(bmi_dec)

Risk Prediction Interval: Observed Followup

Number of study subjects: 5285

Number of cases: 1251

Follow-up time (years) [mean,range]: [9.706, (5,13)]

Baseline age (years) [mean,range]: [62.556, (50,72)]

Absolute Risk Calibration

     Hosmer and Lemeshow goodness of fit (GOF) test for Absolute Risk

data: observed.frequency, expected.frequency

Chisquare = 25.925, df = 10, p-value = 0.003843

Relative Risk Calibration

     Goodness of fit (GOF) test for Relative Risk

data: observed.frequency, expected.frequency

Chisquare = 35.528, df = 9, p-value = 4.807e-05

Model Discrimination

Estimate of AUC: 0.587

95% CI of AUC: (0.568,0.605)

Overall Expected to Observed Ratio

Estimate: 0.967

95% CI: (0.908,1.03)

**Fig 2:**
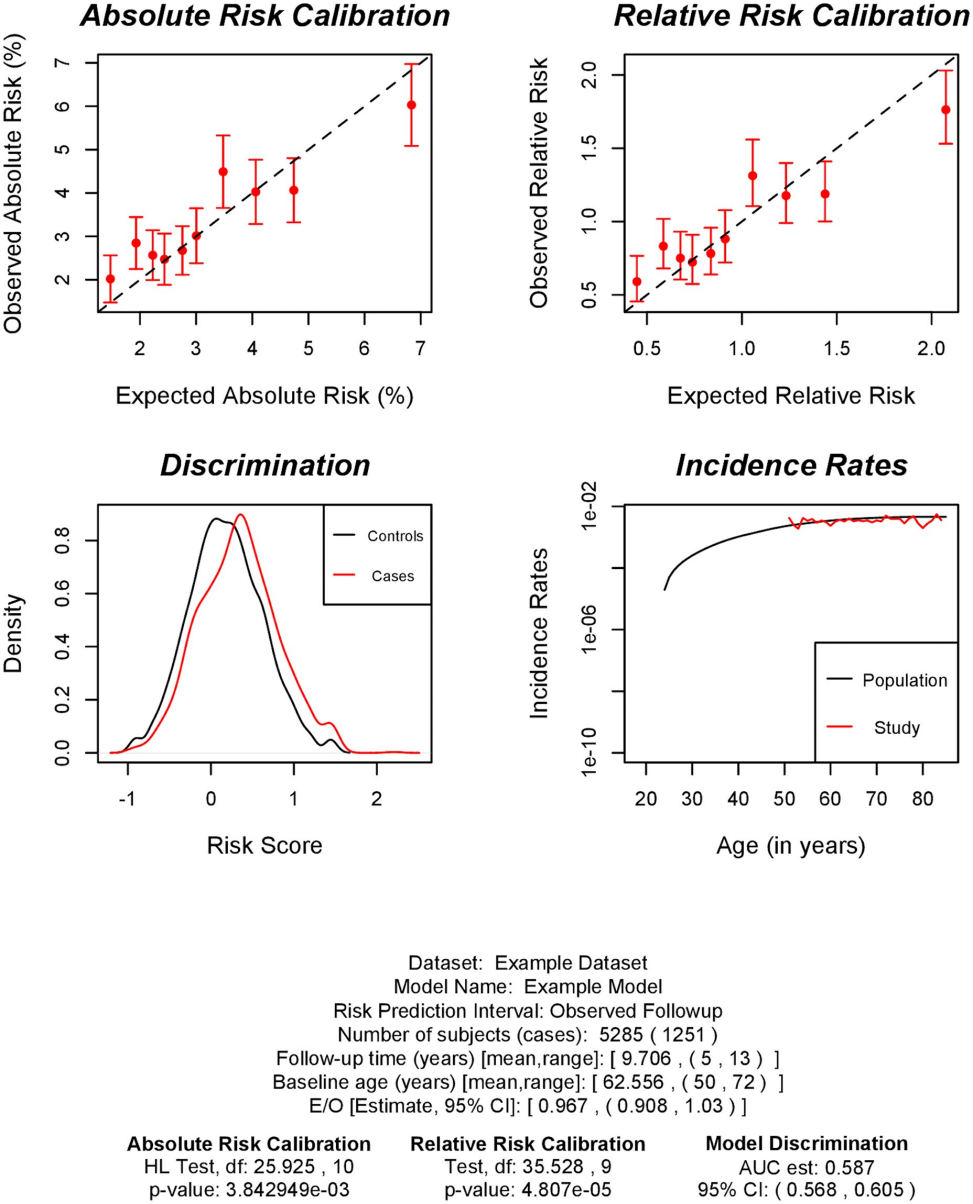
Plots showing model validation results. The top left panel shows calibration results for absolute risk; the top right panel shows calibration for relative risk with respect to the average risk in the validation study; the bottom left panel shows the distribution of risk score in cases (red) and controls (black); the bottom right panel shows the population incidence rates (black) and the incidence rates estimated in validation study (red).

We can also produce an useful plot using the command plot(output) showing the validation results (Fig 2).

The goodness-of-fit test results indicate poor model calibration overall and it is driven by some risk categories. The example dataset is simulated from a Cox regression model where the baseline hazard has a particular Weibull distribution (details in Appendix). In the validation analysis, we estimate the baseline hazard using the procedure in the paper that uses information from the marginal age-specific breast cancer incidence rates from the Surveillance, Epidemiology, and End Results (SEER) database of the National Cancer Institute. The miscalibration in the model is expected due to mismatch between the SEER incidence rates with those generated from the Weibull model. We have investigated through simulations that using the Weibull model based incidence rates the model shows good calibration on an average. The example dataset, which was made previously available in iCARE, still serves the purpose of illustrating the working of the validation component of the package.

### Additional options

The iCARE software provides several advanced options as well. For example, model.ref.dataset.weights allows the user to optionally specify a vector of weights for each row in the reference dataset. Whenever any averaging is performed over the reference dataset, such as in the case of missing covariates for prediction, a weighted average is applied using the provided sampling weights.

Using the computeAbsoluteRiskSplitInterval function a user can specify that the absolute risk interval be computed in two parts, using two different sets of parameters. This allows the proportional hazards assumption to be relaxed to some extent, by allowing the relationship between risk factors and the outcome to vary over time. For example, it is well documented that the relationships between certain risk factors, such as body mass index, and breast cancer are different among premenopausal and postmenopausal women. Using computeAbsoluteRiskSplitInterval, users can specify a different set of relative risks through the input parameters model.log.RR and model.log.RR2 for use prior to and after a cutpoint of age 50 years, the median age of menopause. This function is also useful when the distribution of risk factors varies with age.

In addition to returning risk estimates for the specified profiles, the iCARE functions can optionally return the absolute risks for the reference dataset as well if return.refs.risk = TRUE. The risk scores, or *β*^*T*^ *Z*_*i*_, for the covariate profiles can be obtained by setting the parameter return.lp = TRUE. For individuals where there is missing data in covariate profile *Z*, the reported linear predictor is the average of the full linear predictors of all referent subjects in the matching strata based on the approach described earlier to handle missing covariate data.

## Conclusion

The iCARE package is a new tool for building, validating and applying absolute risk models by synthesizing data sources on key model parameters. The tool standardizes methodology and gives researchers the ability to easily update and share absolute risk models, and to evaluate the public health implications of etiologic findings by translating relative risks onto the absolute risk scale. The package incorporates calibration to population-based age-specific disease incidence rates and handling of missing data by leveraging a reference dataset of risk factors for the population of interest. Through its handling of missing data and the ability to incorporate SNP information based on published estimates, the tool gives researchers the ability to easily handle data analytic issues that are likely to arise in practice when building absolute risk models for public health. The package also incorporates standardized methods and tools for validating an absolute risk prediction model in independent studies: a step that is critical for clinical application of risk models (e.g., risk based screening). In this article, we have described the methodology underlying this new tool and illustrated its use with examples by building and validating absolute risk models for breast cancer. This tool can be ultimately used for clinical decision making. In that context, while the current package can run in the background, there needs to be more user friendly web or mobile application based interfaces that could be easily accessed by clinicians or general public. The package, in its current form, will be most useful for public health researchers.

## Appendix

### Variance of category specific absolute risk

Here we derive the influence function based variance formula for $\text{Var}[{\hat{p}}_{o,c}]$ in a nested case-control study setting, where ${\hat{p}}_{o,c}$ is the observed proportion of cases in the cateogry *c*. We denote by *p*_*c*_, the probability limit of ${\hat{p}}_{o,c}$. This estimator can be obtained by solving the estimating equation based on the total number of subjects (*N*) in the cohort:

$$\begin{array}{c} \sum_{i=1}^{N}\frac{I_{i}}{\pi (Y_{i},Z_{i})}I\left({\tilde{R}}_{i}=c\right)\left(Y_{i}-p_{c}\right)=0 \end{array}$$

The influence function representation of this estimator is given by:

$$\begin{array}{c} \left({\hat{p}}_{o,c}-p_{c}\right)\approx \frac{1}{NP[{\tilde{R}}_{i}=c]}\sum_{i=1}^{N}\frac{I_{i}}{\pi (Y_{i},Z_{i})}I\left({\tilde{R}}_{i}=c\right)\left(Y_{i}-p_{c}\right) \end{array}$$

Based on this influence function representation, the asymptotic variance formula is given by:

$$\begin{array}{c} \text{Var}\left[{\hat{p}}_{o,c}\right]\approx \frac{1}{N\text{Pr}[{\tilde{R}}_{i}=c]}(p_{c}\left(1-p_{c}\right)+\text{E}[\frac{{\left(Y-p_{c}\right)}^{2}\left(1-\pi \left(Y,Z\right)\right)}{\pi (Y,Z)}|{\tilde{R}}_{i}=c]) \end{array}$$

The variance formula includes a binomial variance term and a correction factor that comes in due to the adjustment for non-random sampling of subjects in the nested case-control study. For the full cohort setting, the second term will be zero and only the first term contributes to the $\text{Var}[{\hat{p}}_{o,c}]$.

### Variance of category specific relative risk

The category specific relative risk is defined as the ratio of the category specific absolute risk and the average absolute risk in the validation study. The categories include approximately equal number of subjects. Hence, the average absolute risk can be approximated by the average of the absolute risk over the categories. We assume that the vector of the centered and scaled category specific absolute risks follows a multivariate normal distribution with mean zero and a diagonal variance-covariance matrix (Σ_*AR*_) with the diagonal entries given by the category specific variance of absolute risk. The vector of category specific relative risks is a smooth function of the vector of category specific absolute risks. Hence we implement a Delta method to compute the variance-covariance matrix (Σ_*RR*_) of the vector of category specific relative risks.

We denote the category specific observed relative risk to be ${\hat{RR}}_{o,c}$ and the category specific predicted relative risk to be ${\hat{RR}}_{e,c}$. We further denote, ${\hat{RR}}_{o}={\left({\hat{RR}}_{o,1},\ldots ,{\hat{RR}}_{o,C-1}\right)}^{T}$, ${\hat{RR}}_{e}={\left({\hat{RR}}_{e,1},\ldots ,{\hat{RR}}_{e,C-1}\right)}^{T}$ and ${\tilde{\Sigma }}_{RR}$ denote the corresponding (*C* − 1) by (*C* − 1) submatrix of Σ_*RR*_. We implement a test for relative risk calibration using the test statistic ${\left({\hat{RR}}_{o}-{\hat{RR}}_{e}\right)}^{T}{\tilde{\Sigma }}_{RR}^{-1}\left({\hat{RR}}_{o}-{\hat{RR}}_{e}\right)$ that can be approximated by a chisquare distribution with (*C* − 1) degrees of freedom.

### Variance of Area Under the Curve (AUC)

We denote by *I*_1_(*I*_0_), the indicator of inclusion of a case(control) in the cohort study to the nested case-control sample. Let *n*_1_(*n*_0_) be the number of cases (controls) in the cohort. The inverse probability weighted estimator $\hat{\delta }$ is obtained by solving the estimating equation: $\sum_{i=1}^{n_{1}}\sum_{j=1}^{n_{0}}\frac{I_{1i}I_{0j}}{\pi \left(1,Z_{i}\right)\pi \left(0,Z_{j}\right)}(I\left(R_{1i}>R_{0j}\right)-\delta )=0$. The influence function representation of this estimator is given by: $\left(\hat{\delta }-\delta \right)\approx \frac{1}{n_{1}}\sum_{i=1}^{n_{1}}\frac{I_{1i}}{\pi (1,Z_{i})}({\text{E}}_{R_{0}}\left[I\left(R_{1i}>R_{0}\right)\right]-\delta )+\frac{1}{n_{0}}\sum_{j=1}^{n_{0}}\frac{I_{0j}}{\pi (0,Z_{j})}\left({\text{E}}_{R_{1}}\left[I\left(R_{1}>R_{0j}\right)\right]-\delta \right)$. Based on this representation, the asymptotic variance formula is given by:

$$\begin{array}{ccc} \text{Var}[\hat{\delta }] & \approx & \frac{1}{n_{1}}(\text{Var}\left[{\text{E}}_{R_{0}}\left[I\left(R_{1}>R_{0}\right)\right]\right]\\ & & \;+\text{E}[\frac{{\left({\text{E}}_{R_{0}}\left[I\left(R_{1}>R_{0}\right)\right]-\delta \right)}^{2}\left(1-\pi \left(1,Z\right)\right)}{\pi (1,Z)}])\\ & & +\frac{1}{n_{0}}(\text{Var}\left[{\text{E}}_{R_{1}}\left[I\left(R_{1}>R_{0}\right)\right]\right]\\ & & \;+\text{E}[\frac{{\left({\text{E}}_{R_{1}}\left[I\left(R_{1}>R_{0}\right)\right]-\delta \right)}^{2}\left(1-\pi \left(0,Z\right)\right)}{\pi (0,Z)}]) \end{array}$$

### Simulation study design for illustration of model validation

We simulate data for a cohort study with 50,000 subjects with information on age of study entry, age of study exit, time to disease onset, disease status, classical risk factors and the 72 breast cancer associated SNPs in bc_72_snps. The classical risk factors are generated by resampling the reference dataset (described in Example 2 in the manuscript) to create a dataset with 50,000 subjects. We generate the 72 breast cancer associated SNPs using the allele frequencies available in bc_72_snps and accounting for correlation with family history (details described in manuscript). We use the log relative risks for classical risk factors given in bc_model_log_or. For the SNPs, we use the log odds ratios given in bc_72_snps. We generate an age of disease onset from a Cox model that assumes multiplicative association of the risk factors on disease risk. The baseline hazard function of the Cox model is generated from a Weibull distribution with parameters with hazard function λ_0_(*t*) = λ*γt*^*γ*−1^ with λ = 0.9 × 10^−6^ and *γ* = 2.15. We generate age of study entry from a discrete uniform distribution in the range 50 years to 72 years. We also generate a length of observed follow-up from a discrete uniform distribution in the range 5 years to 13 years.

After we generate the full cohort data, we select a case-control sample nested within the cohort and assume that data on the 72 SNPs are available only on this sub-sample. We assume a logistic regression model of selection depending on case/control status, age of study entry and observed follow-up and compute the selection probabilities using the following code:

selection.model.params = c(-6.52,8.47,0.05,0.11,-0.09,0.04)

selection.model.matrix = model.matrix(~observed.outcome *

                    (study.entry.age + observed.followup),

                  data = validation.cohort.data)

selection.exp.lin.pred = exp(selection.model.matrix %*%

                    selection.model.params)

selection.prob = (selection.exp.lin.pred/(1 + selection.exp.lin.pred))

In the above code, the selection.model.params object gives the log-odds ratios associated with the covariates in the selection model. The selection probabilities are used to generate a binary inclusion (yes = 1, no = 0) variable from a Bernoulli distribution. All the subjects with inclusion taking value 1 are included in the nested case-control sample. Both the full cohort and nested case-control datasets have been made available with iCARE under the names validation.cohort.data and validation.nested.case.control.data, respectively.

## Supporting information

S1 Data (ZIP)

## References

[1] Gail MH, Brinton LA, Bfyar DP, Donald K, Green SB, Schairer C, et al Projecting Individualized Probabilities of Developing Breast Cancer for White Females Who Are Being Examined Annually. Journal Of The National Cancer Institute. 1989; p. 1879–1886. 10.1093/jnci/81.24.1879 2593165

[2] Pfeiffer RM, Gail MH. Absolute Risk: Methods and Applications in Clinical Management and Public Health. Chapman and Hall/CRC; 2017.

[3] Jackson R. Guidelines on preventing cardiovascular disease in clinical practice. BMJ. 2000;320(7236):659–661. 10.1136/bmj.320.7236.659 10710556 PMC1117692

[4] Jackson R, Lawes CM, Bennett DA, Milne RJ, Rodgers A. Treatment with Drugs to Lower Blood Pressure and Blood Cholesterol Based on an Individual’s Absolute Cardiovascular Risk. Lancet. 2005;365(9457):434–441. 15680460 10.1016/S0140-6736(05)17833-7

[5] Pharoah PDP, Antoniou AC, Easton DF, Ponder BAJ. Polygenes, Risk Prediction, and Targeted Prevention of Breast Cancer. New England Journal of Medicine. 2008;358(26):2796–2803. 10.1056/NEJMsa0708739 18579814

[6] Gail MH. Personalized Estimates of Breast Cancer Risk in Clinical Practice and Public Health. Statistics in Medicine. 2011;30(10):1090–1104. 10.1002/sim.4187 21337591 PMC3079423

[7] Grundy SM. Primary prevention of coronary heart disease: integrating risk assessment with intervention. Circulation. 1999;100(9):988–998. 10.1161/01.cir.100.9.988 10468531

[8] Gail MH. The Estimation and Use of Absolute Risk for Weighing the Risks and Benefits of Selective Estrogen Receptor Modulators for Preventing Breast Cancer. Annals of the New York Academy of Sciences. 2001;949(1):286–291. 10.1111/j.1749-6632.2001.tb04034.x 11795364

[9] Murray CJ, Lauer JA, Hutubessy RC, Niessen L, Tomijima N, Rodgers A, et al Effectiveness and costs of interventions to lower systolic blood pressure and cholesterol: a global and regional analysis on reduction of cardiovascular-disease risk. Lancet. 2003;361(9359):717–725. 10.1016/S0140-6736(03)12655-4 12620735

[10] R Development Core Team. R: A Language and Environment for Statistical Computing. R Foundation for Statistical Computing, Vienna, Austria. 2010.

[11] Cox DR. Regression Models and Life-Tables. Journal of the Royal Statistical Society Series B (Methodological). 1972;34(2):pp. 187–220. 10.1111/j.2517-6161.1972.tb00899.x

[12] Prentice RL, Breslow NE. Retrospective studies and failure time models. Biometrika. 1978;65(1):153–158. 10.1093/biomet/65.1.153

[13] Howlader N, Noone A, Krapcho M, Neyman N, Aminou R, Waldron W, et al. SEER Cancer Statistics Review, 1975-2008. National Cancer Institute. 2011.

[14] Rubin DB. In: Procedures with Ignorable Nonresponse. John Wiley & Sons, Inc; 2008 p. 154–201.

[15] Hosmer DW, Hosmer T, Le Cessie S, Lemeshow S. A comparison of goodness-of-fit tests for the logistic regression model. Statistics in Medicine. 1997;16(9):965–980. 10.1002/(sici)1097-0258(19970515)16:9<965::aid-sim509>3.0.co;2-o 9160492

[16] DeLong ER, DeLong DM, Clarke-Pearson DL. Comparing the Areas Under Two or More Correlated Receiver Operating Characteristic Curves: A Nonparametric Approach. Biometrics. 1988;44(3):837–845. 10.2307/2531595 3203132

[17] Cai T, Zheng Y. Nonparametric Evaluation of Biomarker Accuracy Under Nested Case-control Studies. Journal of the American Statistical Association. 2011;106(494):569–580. 10.1198/jasa.2011.tm09807 22844169 PMC3404857

[18] Cai T, Zheng Y. Evaluating Prognostic Accuracy of Biomarkers in Nested Case-control Studies. Biostatistics. 2012;13(1):89–100. 10.1093/biostatistics/kxr021 21856652 PMC3276269

[19] Zheng Y, Cai T, Pepe MS. Adopting Nested Case-control Quota Sampling Designs for the Evaluation of Risk Markers. Lifetime Data Analysis. 2013;19(4):568–588. 23807695 10.1007/s10985-013-9270-8 PMC3903399

[20] Zhou QM, Zheng Y, Cai T. Assessment of Biomarkers for Risk Prediction with Nested Case-control Studies. Clinical Trials. 2013;10(5):677–679. 10.1177/1740774513498321 24013405 PMC3800233

[21] Yao W, Li Z, Graubard BI. Estimation of ROC Curve with Complex Survey Data. Statistics in Medicine. 2015;34(8):1293–1303. 10.1002/sim.6405 25546290 PMC4355032

[22] Pal Choudhury P, Chaturvedi AK, Chatterjee N. Evaluating discriminatory accuracy of models using partial risk-scores in two-phase studies. arXiv:171004379. 2017.

[23] Tyrer J, Duffy SW, Cuzick J. A breast cancer prediction model incorporating familial and personal risk factors. Statistics in Medicine. 2004;23(7):1111–1130. 10.1002/sim.1668 15057881

[24] Cuzick J. IBIS: Breast Cancer Risk Evaluation Tool. Package Version 80. 2017.

[25] Antoniou AC, Pharoah PPD, Smith P, Easton DF. The BOADICEA model of genetic susceptibility to breast and ovarian cancer. British Journal of Cancer. 2004;91(8):1580–1590. 10.1038/sj.bjc.6602175 15381934 PMC2409934

[26] Antoniou AC, et al The BOADICEA model of genetic susceptibility to breast and ovarian cancers: updates and extensions. British Journal of Cancer. 2008;98(8):1457–1466. 10.1038/sj.bjc.6604305 18349832 PMC2361716

[27] Lee AJ, Cunningham AP, Kuchenbaecker KB, Mavaddat N, Easton DF, Antoniou AC. BOADICEA breast cancer risk prediction model: updates to cancer incidences, tumour pathology and web interface. British Journal of Cancer. 2014;110(2):535–545. 10.1038/bjc.2013.730 24346285 PMC3899766

[28] Cunningham A, Antoniou A. Breast and Ovarian Analysis of Disease Incidence and Carrier Estimation Algorithm (BOADICEA). Package Version 30. 2018.

[29] Gerds TA, Scheike TH, Blanche P, Ozenne B. riskRegression: Risk Regression Models and Prediction Scores for Survival Analysis with Competing Risks. R package Version 143. 2017.

[30] Gerds TA, Scheike TH, Anderson PK. Absolute Risk Regression for Competing Risks: Interpretation, Link Functions, and Prediction. Statistics in Medicine. 2012;31(29):3921–3930. 10.1002/sim.5459 22865706 PMC4547456

[31] Benichou J, Gail MH. Estimates of Absolute Cause-specific Risk in Cohort Studies. Biometrics. 1990; p. 813–826. 10.2307/2532098 2242416

[32] Kundu S, Aulchenko YS, Blanche P, Janssens ACJW. PredictABEL: Assessment of Risk Prediction Models. R package Version 12-2. 2015.

[33] Chen S, Wang W, Broman KW, Katki HA, Parmigiani G. BayesMendel: An R environment for Mendelian Risk Prediction. Statistical Applications in Genetics and Molecular Biology. 2004;3(1):1–19. 10.2202/1544-6115.1063 PMC2274007 16646800

[34] Zhang F. BCRA: Breast Cancer Risk Assessment. R package Version 20. 2018.

[35] Costantino JP, Gail MH, Pee D, Anderson S, Redmond CK, Benichou J, et al Validation studies for models projecting the risk of invasive and total breast cancer incidence. Journal of the National Cancer Institute. 1999;91(18):1541–1548. 10.1093/jnci/91.18.1541 10491430

[36] Gail MH, Costantino JP, Pee D, Bondy M, Newman L, Selvan M, et al Projecting individualized absolute invasive breast cancer risk in African American women. Journal of the National Cancer Institute. 2007;99(23):1782–1792. 10.1093/jnci/djm223 18042936

[37] Matsuno RK, Costantino JP, Ziegler RG, Anderson GL, Li H, Pee D, et al Projecting individualized absolute invasive breast cancer risk in Asian and Pacific Islander American women. Journal of the National Cancer Institute. 2011;103(12):951–961. 10.1093/jnci/djr154 21562243 PMC3119648

[38] Banegas MP, John EM, Slattery ML, Gomez SL, Yu M, LaCroix AZ, et al Projecting individualized absolute invasive breast cancer risk in US Hispanic women. Journal of the National Cancer Institute. 2016;109(2):djw215 10.1093/jnci/djw215 PMC5174188 28003316

[39] Gong G. rmap: Risk Model Assessment Package. R package v-00301. 2016.

[40] Gong G, Quante AS, Terry MB, Whittemore AS. Assessing the Goodness of Fit of Personal Risk Models. Statistics in Medicine. 2014;33(18):3179–3190. 10.1002/sim.6176 24753038 PMC4362710

[41] Whittemore AS, Halpern J. Two-stage Sampling Designs for External Validation of Personal Risk Models. Statistical Methods in Medical Research. 2016;25(4):1313–1329. 10.1177/0962280213480420 23592716 PMC3971015

[42] Michailidou K, Lindström S, Dennis J, Beesley J, Hui S, Kar S, et al Association analysis identifies 65 new breast cancer risk loci. Nature. 2017;551:92–94. 10.1038/nature24284 29059683 PMC5798588

[43] National Center for Health Statistics (NCHS). Underlying Cause of Death 1999-2011 on CDC WONDER Online Database, released 2014. Data are from the Multiple Cause of Death Files, 1999-2011, as compiled from data provided by the 57 vital statistics jurisdictions through the Vital Statistics Cooperative Program.; 2014. Available from: http://wonder.cdc.gov/ucd-icd10.html.

[44] Maas P, Barrdahl M, Joshi AD, Auer PL, Gaudet MM, Milne RL, et al Breast Cancer Risk From Modifiable and Nonmodifiable Risk Factors Among White Women in the United States. JAMA Oncology. 2016;2(10):1295–1302. 10.1001/jamaoncol.2016.1025 27228256 PMC5719876

[45] Campa D, et al Interactions Between Genetic Variants and Breast Cancer Risk Factors in the Breast and Prostate Cancer Cohort Consortium. Journal of the National Cancer Institute. 2011;103(16):1252–1263. 10.1093/jnci/djr265 21791674 PMC3156803

[46] Joshi AD, et al Additive Interactions Between Susceptibility Single-Nucleotide Polymorphisms Identified in Genome-Wide Association Studies and Breast Cancer Risk Factors in the Breast and Prostate Cancer Cohort Consortium. American Journal of Epidemiology. 2014;180(10):1018–1027. 10.1093/aje/kwu214 25255808 PMC4224360

[47] Maas P, Barrdahl M, Joshi AD, Auer PL, Gaudet MM, Milne RL, et al Breast cancer risk from modifiable and nonmodifiable risk factors among white women in the United States. JAMA Oncology. 2016;2(10):1295–1302. 10.1001/jamaoncol.2016.1025 27228256 PMC5719876

